# Safety and Efficacy of a New Renal Denervation Catheter in Hypertensive Patients in the Absent of Antihypertensive Medications: A Pilot Study

**DOI:** 10.1155/2019/7929706

**Published:** 2019-02-14

**Authors:** Yang Li, Abdul Qadir Nawabi, Yi Feng, Qiming Dai, Genshan Ma, Naifeng Liu

**Affiliations:** ^1^Department of Cardiology, Zhongda Hospital, Medical School, Southeast University, 87 Dingjia Bridge Road, Nanjing 210009, China; ^2^Department of Cardiology, Lishui People's Hospital, 86 Chongwen Road, Nanjing 211200, China

## Abstract

**Aim:**

The aim of present study was to determine the safety and efficacy of a new renal artery denervation system for treatment of hypertensive patients.

**Methods:**

Hypertensive patients with mean office systolic blood pressure ≥150mmHg and ≤180mmHg or an average of 24-hour ambulatory systolic blood pressure ≥145mmHg and ≤170mmHg after stopping hypertensive medications for 2 weeks or more were enrolled to undergo renal denervation (RDN) using a new RDN system. Changes in office blood pressure and mean 24-hour ambulatory blood pressure and safety were assessed after 6 months.

**Results:**

Fifteen patients underwent RDN and followed up for 6 months. At the 6-month follow-up, office systolic blood pressure decreased 11.5±9.9mmHg (P<0.01) and office diastolic blood pressure decreased 6.9±4.8mmHg (P<0.01); mean 24-hour ambulatory systolic blood pressure decreased 7.5±7.7mmHg (P<0.05) and mean 24-hour diastolic blood pressure decreased 3.3±4.7mmHg (P>0.05) compared to baseline values. There were no serious RDN-related adverse events during follow-up.

**Conclusion:**

Our results demonstrate that the new RDN system is safe and could significantly reduce blood pressure in hypertensive patients in the absence of antihypertensive medications. This trial is registered with ChiCTR1800017815.

## 1. Introduction

Hyperactivity of the sympathetic nervous system contributes an important role in the pathophysiology of hypertension. Renal denervation (RDN) is a new interventional treatment for resistant hypertension in recent years [1–4]. Recent clinical studies have shown that there are some controversies about the efficacy of RDN in the treatment of hypertension [5–7]. Ablation instruments are important quality assurances for RDN. Improvements in ablation catheters may further improve the quality of the procedure and reduce the operator's operational difficulty, thus potentially ensuring the efficacy of the procedure. This prospective, single-center, self-controlled study was designed to evaluate the efficacy and safety of a new RDN system [(ablator no.: GL-06E15WA, ablation catheter no: GL-6w (12mm), Shanghai Golden Leaf Medtech Company, Shanghai, China] in hypertensive patients without antihypertensive medication.

## 2. Methods

### 2.1. Subject Selection

Inclusion criteria are (1) age >18 and ≤75 years; (2) mean office systolic blood pressure ≥150mmHg and ≤180mmHg; or an average of 24-hour ambulatory systolic blood pressure ≥145mmHg and ≤170mmHg after stopping hypertensive medications for 2 weeks or more; (3) renal artery length ≥20mm. Exclusion criteria are (1) secondary hypertension; (2) glomerular filtration rate(GFR) <40mL/min; (3) unilateral or bilateral renal artery anatomy; (4) office SBP >180mmHg and/or diastolic blood pressure >110 mmHg after stopping hypertensive medication during the enrollment period. The study was approved by the Zhongda Hospital Ethics Committee of Southeast University (ethical approval number: 2015ZDSYLL077.0). NCT no. is ChiCTR1800017814. All enrolled patients signed informed consent.

### 2.2. RDN Procedure

**Figure 1:**
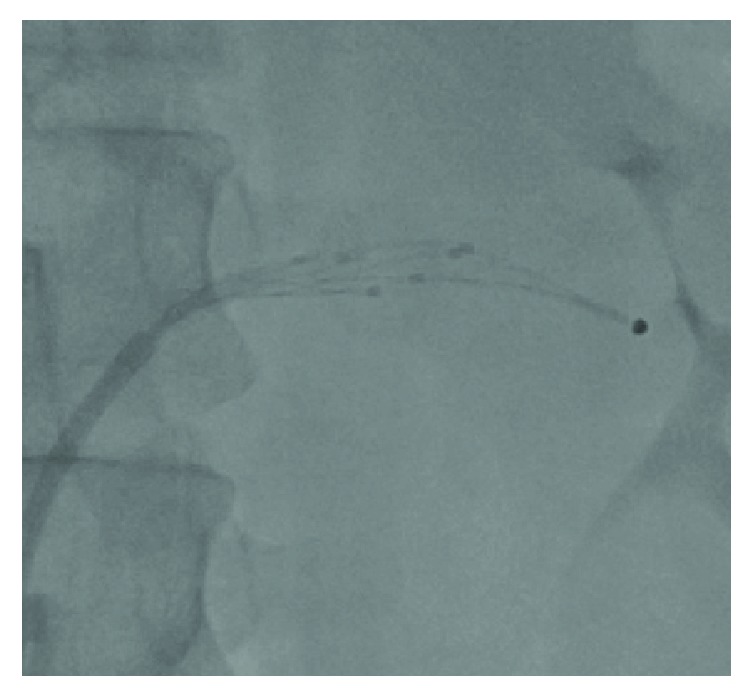
Angiographic image of the renal denervation catheter applying circumferential ablations.

RDN was performed by using the newly developed RDN system (GL-06E15WA ablator and GL-6W ablation catheter) developed by Shanghai Golden Leaf Medtech Company, Shanghai, China. The major features of this RND system are as follows: this system is easy to handle and supplies a 360° circular ablation with 6 electrodes, without affecting renal artery blood flow and the impact of respiratory movement on ablation is minimal. RDN procedure was performed as previously described [8]. Patients were taken to the catheterization laboratory to undergo the RDN procedure using conscious sedation. The ablation catheter should be advanced to the place where the electrode tip was fully visible in the renal artery and then pushed the electrode expansion button on the catheter handle to fit the electrode to the vessel wall (Figure 1) and then began the adherent diagnosis, observing the temperature change of the target ablation locations. The temperature was gradually increased to prove that adherence to the wall is suitable. The ablation parameter is set to 60°C and the ablation time is 120 seconds per point. After completion of 6 points ablation, the electrode expansion button was released to shrink the electrode tip and slowly returned to the guide sheath. Fentanyl citrate 1-2ug/kg.h was maintained intravenously for analgesia treatment. Postoperative antiplatelet therapy (aspirin 100mg po qd; clopidogrel 75mg po qd) was applied for 4 weeks after RDN.

*Study Endpoints*. Mean office blood pressure and 24-hour ambulatory blood pressure, remote blood pressure monitoring data, and complications at 6 months were followed up. Safety: the occurrence of study-related adverse events during the trial, especially renal function indicators and renal artery complications. Adverse events or serious adverse events that may occur during or after the procedure: renal artery stenosis, renal artery dissection, thromboembolism, artery puncture site complications, arteriovenous fistula, sepsis, and other possible adverse reactions during the period [2].

### 2.3. Statistical Analysis

The continuous data were expressed as mean±standard deviation. Differences from baseline to the 6-month follow-up assessment were tested with the use of paired t-tests. P <0.05 was considered as statistically significant.

## 3. Result

### 3.1. Baseline Characteristics

A total of 15 patients (14 male) underwent RDN and all finished the 6-month follow-up. The mean age was 39.0±7.0 years, average heart rate was 70±2.6 beats/min, and the average GFR was 127.8±24.5 mL/min.

### 3.2. Efficacy

The office systolic blood pressure was 158.2±6.4mmHg at baseline, 146.7±11.6mmHg at 6 months after RDN. The office diastolic blood pressure was 100.3±8.8mmHg at baseline and 93.4±7.2mmHg at 6 months after RDN. Office systolic blood pressure decreased 11.5±9.9mmHg (P<0.01) and diastolic blood pressure decreased 6.9±4.8mmHg (P<0.01) at 6 months post RDN compared to baseline levels (Table 1).

The mean 24-hour ambulatory systolic blood pressure was 154.5±10.7mmHg at baseline and 147.0±12.0 at 6 months after RDN (reduction was -7.5±7.7mmHg compared to baseline, P<0.05). The mean 24-hour ambulatory diastolic blood pressure at baseline was 97.5±8.1mmHg and 94.2±9.2 mmHg at 6 months after RDN (reduction was -7.5±7.7 mmHg compared to baseline, P=0.055)(Table 2).

### 3.3. Safety

Renal function and the average heart rate were similar between baseline and 6-month follow-up. No complications such as renal artery stenosis and renal artery dissection were observed during the 6-month follow-up in this patient cohort.

## 4. Discussion

RDN is an interventional method used for the treatment of refractory hypertension, but the real world and clinical trial efficacy remains controversial. The study of SIMPLICITY HTN-1 and simplicity HTN-2 using SIMPLICITY™ catheter ablation showed that RDN is safe and effective for refractory hypertension[6, 7], but in the prospective, single-blind, randomized, sham-controlled SIMPLICITY HTN-3 trial, RDN failed to significantly reduce blood pressure in patients with refractory hypertension [9]. The following factors might be responsible for the controversial results: (1) insufficient understanding on the working mechanisms of RDN; (2) study design limitations: there is a need to include randomized double-blind, sham-surgery group as control group, and multicenter clinical trials; secondary hypertension should be excluded; the study endpoints should be identical and at best to use the 24-hour ambulatory blood pressure; (3) surgical factors: the learning curve of the operation and the experience of the operators should be comparable and considered on the comparison of various study results. Last but least the efficacy of various ablation catheters used should be taken into account when evaluating the RDN efficacy [10–12]. Moreover, there are also some problems related to the RDN procedures: (1) the degree of ablation on the nerve, temperature on ablation point, and ablation time might all affect the therapeutic effect. Therefore, the RDN system needs to be constantly explored and improved; an ideal RDN ablation catheter should have the following characteristics: (1) the operation should be more simple; (2) the level of dependence on the operator should be reduced to minimum; and (3) the time to achieve the maximum nerve denervation effect should be reduced to the minimum.

The Symplicity™ catheter system is a widely used RDN system in clinical practice now, but this system has several limitations: (1) the catheter has only one electrode, which leads to long ablation time; (2) the selection of vascular ablation site by unipolar electrode is relatively difficult; (3) it is difficult to ablate deep renal sympathetic nerve, since this catheter only possesses low radio frequency power and limited penetration depth. So these deficiencies are likely to be responsible for the uncertainty of clinical trial results. In order to solve the core problem of RND, the latest SPYRAL HTN-OFF MED study used a different innovative Symplicity Spyral multielectrode catheter, which has 4 electrodes, and can ablate 4 locations at the same time from different locations, and preliminary results showed that RDN with this catheter can significantly reduce blood pressure in hypertensive patients in the absence of antihypertensive medications [13, 14].

**Figure 2:**
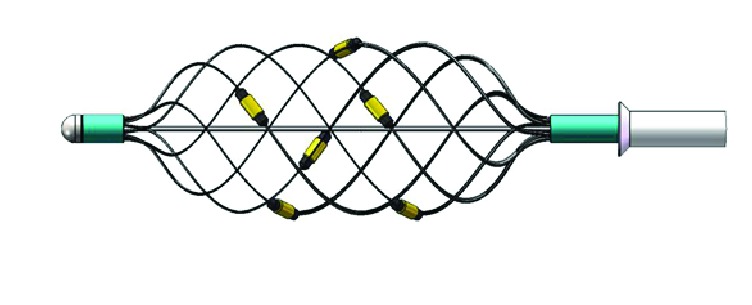
6-point reticular electrodes ablation catheter.

The RDN in our study is achieved through a 6-point reticular electrodes catheter ablation system, which is simple in operation and easy to locate due to the 360° annular ablation design. Its basket-shaped design also guaranteed the impact on renal arterial blood flow, and it was suitable for ablating vessels with different morphologies and sizes. The real time impedance measurement function and temperature monitoring function during RDN procedure are also available to assist the operator to control the efficacy and quality of RDN process (Figure 2).

In conclusion, our preliminary study results show that the new RDN system used in this study is safe and can effectively reduce the blood pressure in primary hypertensive patients in the absence of antihypertensive medication.

## Figures and Tables

**Table 1:** Changes in office blood pressure.

	Systolic blood pressure		Diastolic blood pressure	
	Mean blood pressure	Mean change from baseline	Mean blood pressure	Mean change from baseline
Baseline	158.2±6.4		100.3±8.8	
6 months	146.7±11.6	−11.5±9.9^a^	93.4±7.2	−6.9±4.8^b^

^a^ *P* <0.01; ^b^*P* <0.01 versus baseline.

**Table 2:** Changes in Mean 24-hour ambulatory blood pressure.

	24-hour ambulatory systolic blood pressure		24-hour ambulatory diastolic blood pressure	
	Mean pressure	Mean change from baseline	Mean pressure	Mean change from baseline
Baseline	154.5±10.7		97.5±8.1	
6 months	147.0±12.0	-7.5±7.7^a^	94.2±9.2	-3.3±4.7^b^

^a^ *P* <0.05,^b^*P* =0.055

## Data Availability

Our research data will be shared in the clinical trial management public platform: ResMan.
